# Coculture fermentation processes in wheat sourdough simulation media with *Companilactobacillus crustorum* LMG 23699 and *Wickerhamomyces anomalus* IMDO 010110 reflect their competitiveness and desirable traits for sourdough and sourdough bread production

**DOI:** 10.1128/aem.01325-25

**Published:** 2025-08-12

**Authors:** Inés Pradal, Jasper Kaesemans, Thomas Gettemans, Víctor González-Alonso, Luc De Vuyst

**Affiliations:** 1 Research Group of Industrial Microbiology and Food Biotechnology (IMDO), Faculty of Sciences and Bioengineering Sciences, Vrije Universiteit Brussel (VUB) 70493, Brussels, Belgium; Universita degli Studi di Napoli Federico II, Portici, Italy

**Keywords:** sourdough, starter culture, interaction, microbial consortium, fruity esters

## Abstract

Mixed-strain starter cultures can be used to steer sourdough production and yield sourdoughs containing different volatile organic compounds (VOCs), such as esters with fruity notes. The present study aimed to characterize the fermentation dynamics, including ester production capacity, and investigate the involvement of the latter during potential interactions between a novel lactic acid bacteria (LAB)-yeast consortium composed of *Companilactobacillus crustorum* LMG 23699 and *Wickerhamomyces anomalus* IMDO 010110. Therefore, mono- and co-culture fermentation processes were carried out in a wheat sourdough simulation medium (WSSM) and a modified WSSM that contained ester precursor molecules (mWSSM). The coculture fermentation (CF) processes carried out in WSSM resulted in a faster pH drop and a higher lactic acid production compared with the monoculture ones, which could be of interest in view of the competitive advantage of the LAB-yeast consortium toward background microorganisms. Moreover, these CF processes resulted in higher carbon dioxide production and different VOC profiles, which could be of interest for the production of sourdough breads with desirable specific volumes and flavorful crumbs. Fermentation processes carried out in mWSSM confirmed ethyl lactate biosynthesis by *Coml. crustorum* LMG 23699 and showed a change in the ester profile of the yeast strain. However, the CF processes carried out in this medium showed the vulnerability of the yeast strain toward acidic stress. To conclude, the laboratory fermentation processes of the present study showed the ester production and competitiveness of *Coml. crustorum* LMG 23699 and *W. anomalus* IMDO 010110 as a mixed-strain starter culture for the production of novel sourdoughs and sourdough bread.

**IMPORTANCE:**

Strains of specific species of yeasts and lactic acid bacteria (LAB) often form a stable consortium during sourdough production. Hence, the intentional use of mixed-strain starter cultures composed of such strains can steer sourdough production to obtain, for instance, sourdoughs containing esters with fruity notes. The present study valorized a novel LAB-yeast consortium composed of *Companilactobacillus crustorum* LMG 23699 and *Wickerhamomyces anomalus* IMDO 010110 for the production of such sourdoughs. The application of coculture fermentation processes in wheat sourdough simulation media showed their ester biosynthesis potential and competitiveness. These data will allow the valorization of mixed-strain starter cultures composed of the strains examined.

## INTRODUCTION

Bacteria and yeasts often share the same habitats, and consequently, different interactions are established among specific species and strains (1–4). These interactions play a crucial role in food processing, in particular, flavor formation (5). During food fermentation processes, different types of bacteria-yeast interactions have been described (4, 6), in which cross-feeding and quorum sensing often play an important role (1, 2, 4, 7). However, if the microbial communities of a fermented food ecosystem are stable, exclusion by competition is avoided (4, 8).

The microbiota of a sourdough ecosystem mainly consists of lactic acid bacteria (LAB) and yeasts (9–12). Traditionally, sourdoughs have been produced by spontaneous fermentation using backslopping practices; however, the use of starter cultures steers and increases the success of sourdough production (9, 11). Moreover, a rational selection of starter culture strains can lead to the production of sourdoughs with specific functional characteristics (11, 13). During sourdough production, the impact of environmental and process variables and their changes during continuous propagation upon backslopping enables the stable association of LAB and yeasts (7, 14–17). These associations are generally based on trophic relationships, without competition for carbon or nitrogen sources (16, 18, 19), and for which cross-feeding of nutrients and stress responses play a role too (1, 2, 4, 7). However, competition for carbon sources (20, 21) and neutral or negative interactions (5, 22) have been reported as well. Therefore, understanding how LAB and yeasts interact during sourdough production, including ester biosynthesis potential, will not only shed light on the ecology and mechanisms of their interactions but also allow the development of new mixed-strain starter cultures for food fermentation processes, *in casu* sourdough production.

Apart from the well-known sourdough consortium of *Fructilactobacillus sanfranciscensis* and *Maudiozyma humilis* (19, 20, 23, 24), other LAB-yeast associations have been noted. Examples are those of *Frul. sanfranciscensis* and other yeast species, such as *Saccharomyces cerevisiae* (5, 7, 8, 16, 18, 19, 25, 26), *Maudiozyma exigua* (16), and *Wickerhamomyces anomalus* (25); *Lactiplantibacillus plantarum* and *M. exigua* (27); *Latilactobacillus sakei* and *S. cerevisiae*, *M. humilis*, or *W. anomalus* (25); *Levilactobacillus brevis* and *S. cerevisiae* or *M. exigua* (27); and *Pediococcus pentosaceus* and *S. cerevisiae* (28). Recently, a novel mixed-strain starter culture formed by the LAB strain *Companilactobacillus crustorum* LMG 23699 and the yeast strain *W. anomalus* IMDO 010110 has been reported (29). This homofermentative LAB strain has a fast acidification capacity, based on lactic acid production, and the ability to produce several flavor compounds, such as acetoin (buttery notes; 30, 31) and esters (fruity notes; 32). This yeast strain represents a non-conventional species that has gained attention because of its ester production (33) and antimicrobial activities (34–37). Although these two strains have been successfully used as a mixed-strain starter culture for Type 2 sourdough production (29), the underlying interactions have not been studied in detail. It has indeed to be understood why these strains could form a stable microbial consortium and what their effect could be on mature sourdoughs produced with a mixed-strain starter culture.

The production of esters, condensation products of alcohol and acyl-CoA moieties, by sourdough microorganisms is desired to enrich the bread crumb with fruity notes. Ester production by yeasts is well-known (12, 38), but strains of certain LAB species may produce esters too, although their biosynthesis during sourdough production is limited by the concentrations of the necessary precursor molecules (32). Furthermore, the dependency on strain/species level, the environmental factors, and the fermentation conditions often lead to different sourdough VOC profiles, including ester composition, when starter cultures composed of LAB and yeast strains are used (7, 38, 39). Therefore, the ester biosynthesis capacity by each candidate starter culture strain, as well as the effect of the LAB-yeast interaction on the ester profile produced, should be examined in detail.

The present study aimed to characterize the fermentation dynamics, including ester biosynthesis potential, and the involvement of the latter during interactions between *Coml. crustorum* LMG 23699 and *W. anomalus* IMDO 010110 by performing mono- and co-culture fermentation processes in a wheat sourdough simulation medium (WSSM). Also, it aimed to assess the potential effects of the use of these strains as mixed-strain starter culture on mature sourdoughs and baked goods produced thereof.

## RESULTS

Monoculture fermentation processes were carried out with *Coml. crustorum* LMG 23699 (further referred to as Cc MF processes) and *W. anomalus* IMDO 010110 (Wa MF processes) in both WSSM and a modified WSSM that was supplemented with ester precursor molecules (mWSSM), as well as co-culture fermentation processes (CF) involving both strains.

### Microbial growth courses

**Fig 1:**
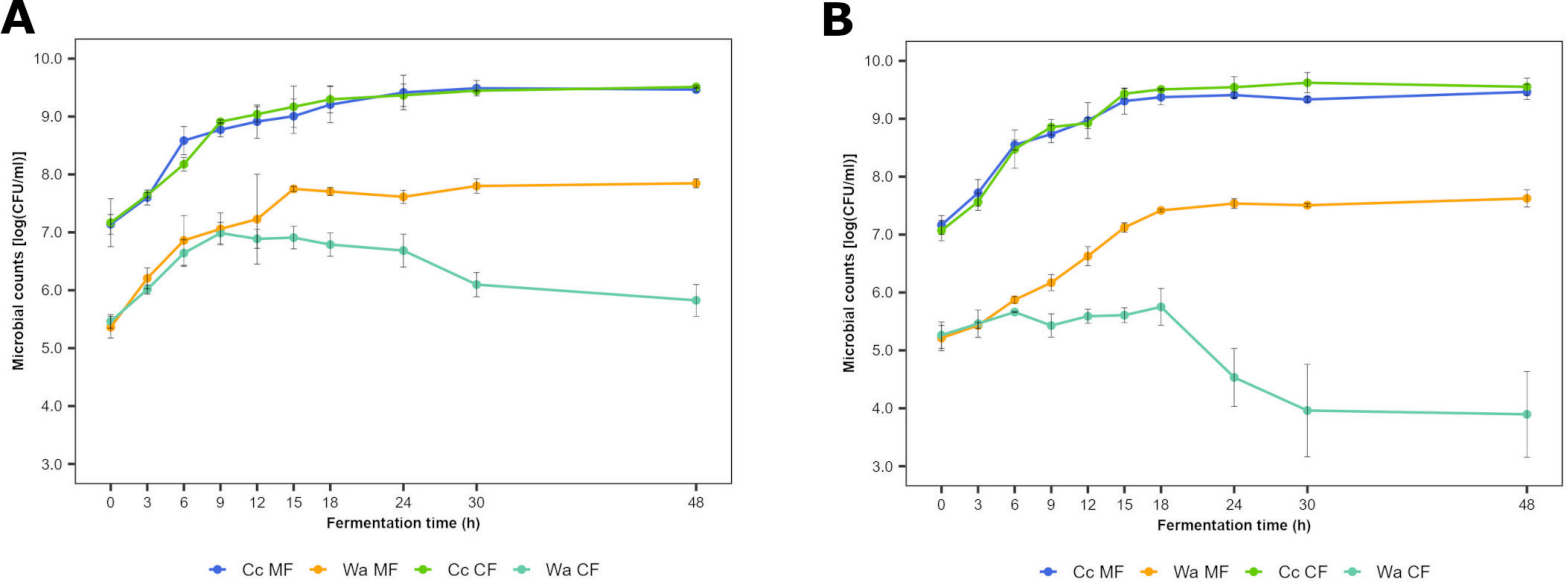
Microbial growth course of *Coml. crustorum* LMG 23699 (Cc) and *W. anomalus* IMDO 010110 (Wa) in WSSM (A) and mWSSM (B) during monoculture (MF) and coculture (CF) fermentation processes, represented as colony-forming units (log [CFU/mL]). The averages and standard deviations of the three fermentation processes are shown.

After inoculation of *Coml. crustorum* LMG 23699 and *W. anomalus* IMDO 010110, the bacterial counts were 7.1 ± 0.2 log (CFU/mL), whereas the yeast counts were 5.3 ± 0.2 log (CFU/mL), hence, obtaining a yeast to LAB ratio of 1:100 at the beginning of the CF processes carried out in both media (Fig. 1A and B). The bacterial counts increased during the first 24 h of fermentation, reaching a plateau at 9.5 ± 0.1 log (CFU/mL) in both media and for both the Cc MF and CF processes, as the same maximum specific growth rate (0.37 h^−1^) was obtained during all fermentation processes. The yeast counts increased during the first 18 h of the Wa MF processes, reaching a plateau at 7.7 ± 0.1 log (CFU/mL) and 7.5 ± 0.1 log (CFU/mL) in WSSM and mWSSM, respectively. The maximum specific growth rate was indeed lower in mWSSM (0.29 h^−1^) than in WSSM (0.33 h^−1^). During the CF processes carried out in WSSM, the yeast counts reached a plateau value of 6.8 ± 0.2 log (CFU/mL) earlier (after 12 h of fermentation) and afterward progressively decreased to 5.9 ± 0.2 log (CFU/mL). The maximum specific growth rate (0.41 h^−1^) was higher than in the Wa MF processes. However, during the CF processes carried out in mWSSM, the yeast counts were stable during 18 h of fermentation and decreased later on to 3.9 ± 0.7 log (CFU/mL).

The values of the optical density at 600 nm (OD_600_) during the exponential growth phase were higher for the CF processes carried out in WSSM than for the MF ones (Fig. S1A). However, after the exponential growth phase, they were lower because of the lower yeast counts reached and their subsequent decrease during the CF processes compared with the Wa MF ones. The cell dry mass (CDM) values followed a similar trend (Fig. S1C). During the fermentation processes carried out in mWSSM, the OD_600_ and CDM values of the CF processes were similar to those of the Cc MF ones, as the yeast counts did not increase during the former processes (Fig. S1B and D).

### pH courses

**Fig 2:**
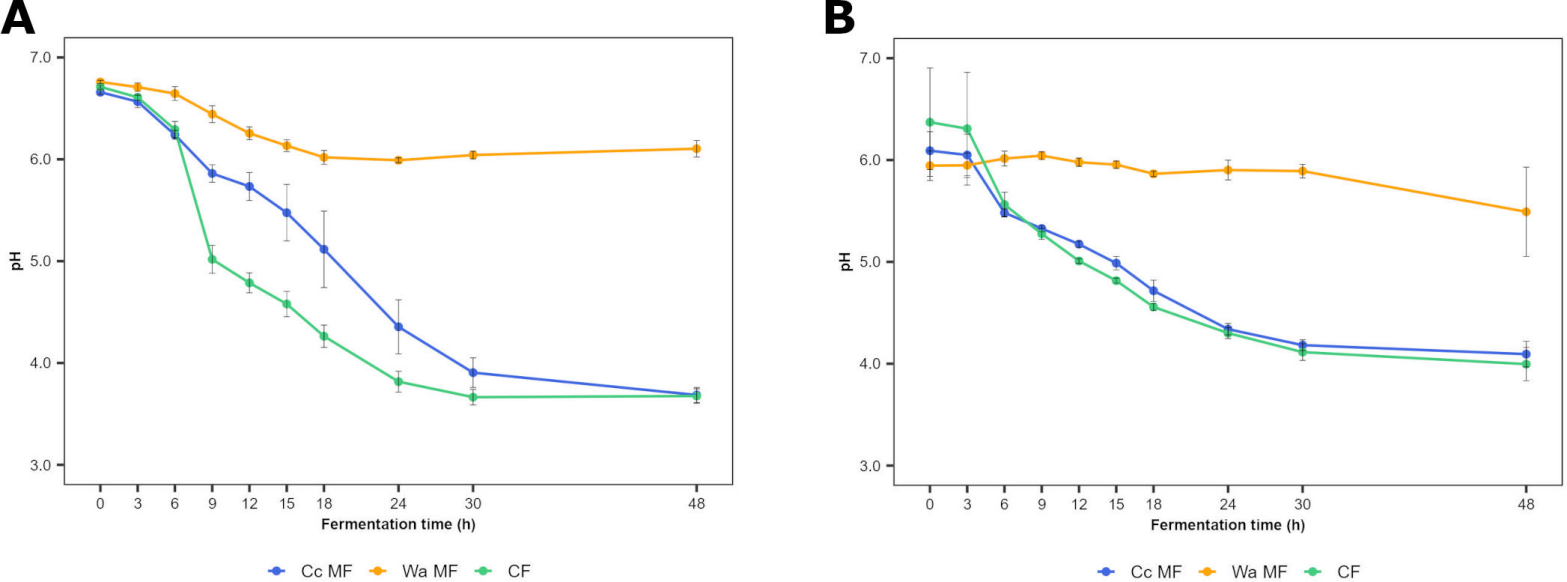
Course of pH values during *Coml. crustorum* LMG 23699 monoculture (Cc MF), *W. anomalus* IMDO 010110 monoculture (Wa MF), and coculture (CF) fermentation processes with both strains carried out in WSSM (**A**) and mWSSM (**B**). The averages and standard deviations of the three fermentation processes are shown.

After inoculation, the pH values of WSSM and mWSSM were 6.7 ± 0.0 and 6.1 ± 0.3, respectively (Fig. 2). During the Wa MF processes, the pH decreased by 10% in both media, reaching values of 6.1 ± 0.1 and 5.5 ± 0.4, respectively. In contrast, during the Cc MF and CF processes, more acidification took place, reaching pH values of 3.7 ± 0.1 and 3.7 ± 0.1, respectively, in WSSM, and 4.1 ± 0.1 and 4.0 ± 0.2, respectively, in mWSSM. Nonetheless, the decrease of the pH values was faster during the CF processes carried out in WSSM than during the Cc MF ones carried out in this medium. In the case of the fermentation processes carried out in mWSSM, the decrease of the pH followed a similar trend during both the Cc MF and the CF processes.

### Substrate consumption and metabolite production and conversion dynamics

#### Carbohydrate concentration dynamics

At the beginning of the fermentation processes, the carbohydrate composition consisted of 24.7 ± 3.7 mM of maltose, 1.4 ± 0.6 mM of sucrose, 3.6 ± 0.6 mM of glucose, and 1.5 ± 0.6 mM of fructose in both media (Fig. S2). During the Cc MF processes, *Coml. crustorum* LMG 23699 did not consume sucrose and used first the monosaccharides glucose and fructose to result in a total depletion after 9 h of fermentation in both media (Fig. S2). During the following 9 h of fermentation and thereafter, the maltose concentrations slowly decreased, followed by a rapid consumption to reach 2.2 ± 2.2 mM after 48 h of fermentation in WSSM and total depletion after 30 h of fermentation in mWSSM.

During the Wa processes carried in WSSM, *W. anomalus* IMDO 010110 broke down sucrose and consumed all glucose and fructose during the first 12 h of fermentation (Fig. S2A). Afterward, *W. anomalus* IMDO 010110 consumed maltose until a total depletion after 30 h of fermentation. A similar trend was found when the fermentation processes were carried out in mWSSM, obtaining a total depletion of sucrose, glucose, and fructose after 18 h of fermentation but a lower maltose consumption (only 20% of the maltose was consumed at the end of the fermentation processes; Fig. S2B).

During the CF processes carried out in WSSM, sucrose, glucose, and fructose were consumed faster than in any of the MF ones, reaching a total depletion at the end of the fermentation processes (Fig. S2A). In contrast, during the CF processes carried out in mWSSM, sucrose concentrations of 0.6 ± 0.3 mM were left at the end of the fermentation processes (Fig. S2B). Maltose was completely consumed in both media (Fig. S2).

#### Lactic acid and other organic acid concentration dynamics

Lactic acid was mainly produced during the fermentation processes in which *Coml. crustorum* LMG 23699 was present (Fig. S3A and B). More lactic acid was produced during the CF processes than during the Cc MF ones in both media (152.8 ± 11.0 *vs* 119.2 ± 0.2 mM, and 162.3 ± 12.3 *vs* 128.7 ± 12.5 mM in WSSM and mWSSM, respectively). The concentrations of lactic acid were on average 9.5 mM higher at the end of the fermentation processes in mWSSM than in WSSM, corresponding with the initial lactic acid concentration of mWSSM. During the Wa MF processes carried out in WSSM, the lactic acid concentrations increased up to 2.0 ± 0.1 mM during the first 15 h of fermentation and later on decreased to 1.0 ± 0.1 mM after 48 h of fermentation (Fig. S3C). In contrast, the lactic acid concentrations remained stable at 8.1 ± 1.7 mM during the Wa MF processes carried out in mWSSM (Fig. S3D).

Citric acid was present at 0.1 ± 0.0 mM during all fermentation processes. The succinic acid concentrations were 0.4 ± 0.1 mM at the beginning of the fermentation processes. During the Cc MF processes carried out in WSSM, succinic acid was depleted after 30 h of fermentation, whereas during the Wa MF and the CF processes, succinic acid accumulated to maximum concentrations of 0.5 ± 0.1 mM after 30 h of fermentation and 0.5 ± 0.2 mM after 48 h of fermentation, respectively. The succinic acid concentrations were stable during the fermentation processes carried out in mWSSM. The concentrations of malic acid and oxalic acid were below the quantification limit during all fermentation processes.

#### Sugar alcohol concentration dynamics

Glycerol was present in concentrations of 1.1 ± 0.2 mM at the start of the fermentation processes (Fig. S4A). Only during the Wa MF processes carried out in WSSM, glycerol accumulated during the exponential growth phase to reach concentrations of 4.0 ± 0.9 mM after 24 h of fermentation. Moreover, arabitol was produced by *W. anomalus* IMDO 010110 during the stationary phase of the Wa MF processes carried out in WSSM, reaching concentrations of 0.1 ± 0.0 mM after 48 h of fermentation.

No sugar alcohols were found during the fermentation processes carried out in mWSSM.

#### Ethanol and acetic acid concentration dynamics

Ethanol accumulated during the Wa MF processes carried out in WSSM, reaching concentrations of 116.1 ± 16.4 mM after 24 h of fermentation (Fig. S4B). A smaller increase in the ethanol concentrations was found during the CF processes in WSSM, reaching values of 31.3 ± 5.7 mM after 24 h of fermentation. At the beginning of the fermentation processes carried out in mWSSM, ethanol and acetic acid were present in concentrations of 143.4 ± 33.9 mM and 93.5 ± 8.0 mM, respectively, corresponding with the concentrations added as ester precursor molecules (Fig. S4C). During the Wa MF processes carried out in mWSSM, ethanol accumulated to concentrations of 195.7 ± 15.5 mM after 24 h of fermentation. Similarly, during the CF processes carried out in mWSSM, the ethanol concentrations increased up to 180.0 ± 7.5 mM after 24 h of fermentation. The acetic acid concentrations were stable during the course of all fermentation processes carried out in mWSSM.

#### Amino acid and biogenic amine concentration dynamics

During the Cc MF processes carried out in WSSM, the free amino acid (AA) concentrations increased from 18 h of fermentation to reach 352.1 ± 109.2 mM after 48 h of fermentation (Fig. S5A). In contrast, for these fermentation processes carried out in mWSSM, the highest AA concentrations (324.4 ± 22.7 mM) were reached after 12 h fermentation (Fig. S5B). During the Wa MF processes carried out in WSSM, there was only a slight increase in the free AA concentrations (Fig. S5A). During the CF processes carried out in WSSM, the AA concentrations increased to reach a maximum of 222.9 ± 26.8 mM. During the Wa MF and CF processes carried out in mWSSM, the AA concentrations barely changed (Fig. S5B).

Specifically during the fermentation processes carried out in WSSM, the alanine and glutamate concentrations increased more during the Cc MF and CF processes than during the Wa MF processes, for which these concentrations were stable. The arginine concentrations decreased during the Cc MF and CF processes but slightly increased during the Wa MF ones. The asparagine concentrations decreased more during the Wa MF and CF processes than during the Cc MF ones. The aspartate concentrations increased more during the MF processes, whereas they did not vary during the CF ones. The gamma-aminobutyric acid (GABA) concentrations increased during the CF processes and varied along the Cc MF ones. Glutamine and ornithine were only quantifiable during the Cc MF processes.

All biogenic amine concentrations were below the quantification limit.

#### Volatile organic compounds

**Fig 3:**
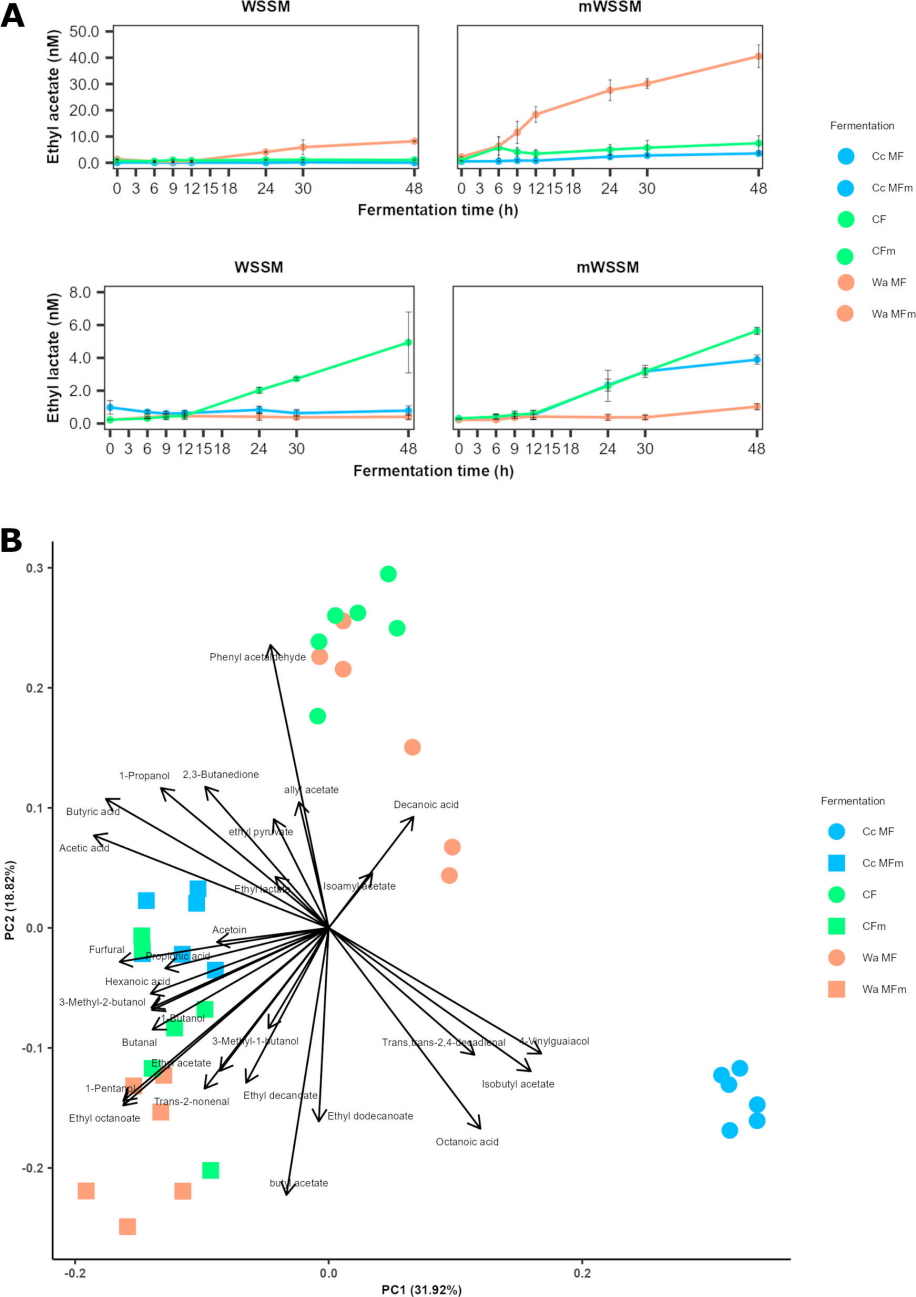
Dynamics of the concentrations of the main esters produced during *Coml. crustorum* LMG 23699 monoculture (Cc MF and Cc MFm), *W. anomalus* IMDO 010110 monoculture (Wa MF and Wa MFm), and coculture (CF and CFm) fermentation processes with both strains carried out in WSSM (Cc MF, Wa MF, and CF) and mWSSM (Cc MFm, Wa MFm, and CFm) (**A**). Principal component analysis of the volatile organic compounds quantified by LI-GC-TQ-MS during *Coml. crustorum* LMG 23699 monoculture (Cc MF and CC MFm), *W. anomalus* IMDO 010110 monoculture (Wa MF and Wa MFm), and coculture (CF and CFm) fermentation processes with both strains carried out in WSSM (Cc MF, Wa MF, and CF) and mWSSM (Cc MFm, Wa MFm, and CFm) (**B**).

The esters isoamyl acetate (banana notes), ethyl acetate (sweet notes), and ethyl lactate (creamy notes) were produced in the highest concentrations, which were above the flavor threshold values for wine and beer (40). The first one reached 1.5 nM during the Wa MF processes carried out in WSSM, whereas its concentration did not vary in any of the other fermentation processes. Ethyl acetate reached a maximum of 8.2 and 40.6 nM during the Wa MF processes carried out in WSSM and mWSSM, respectively, and 3.5 and 7.4 nM in the Cc MF and CF processes carried out in mWSSM, respectively (Fig. 3A). Ethyl lactate was produced in concentrations up to 4.9, 3.9, and 5.6 nM during the CF processes carried out in WSSM, the Cc MF processes carried out in WSSM, and the CF processes carried out in mWSSM, respectively.

Acetoin (buttery notes) was produced during all fermentation processes. During the Cc and Wa MF processes, maximum concentrations of 10.8 and 14.7 mM were produced, respectively, whereas during the CF ones, the acetoin concentrations reached 69.5 mM.

A PCA analysis of the VOC concentrations measured by means of liquid injection gas chromatography with triple-quadrupole tandem mass spectrometry (LI-GC-TQ-MS) revealed two clusters, one formed by the samples of the fermentation processes carried out in WSSM and another one formed by the samples of those carried out in mWSSM (Fig. 3B). The mWSSM cluster was related to several esters as well as to the alcohols and organic acids added as ester precursor molecules. In each cluster, three subclusters could be differentiated, namely, those of the Cc MF, Wa MF, and CF processes. These three subclusters were closer related to each other in the mWSSM cluster than in the WSSM one. For the latter cluster, the Wa MF and CF processes were related to several esters, such as allyl acetate and ethyl pyruvate, as well as phenylacetaldehyde.

#### Carbon dioxide

**Fig 4:**
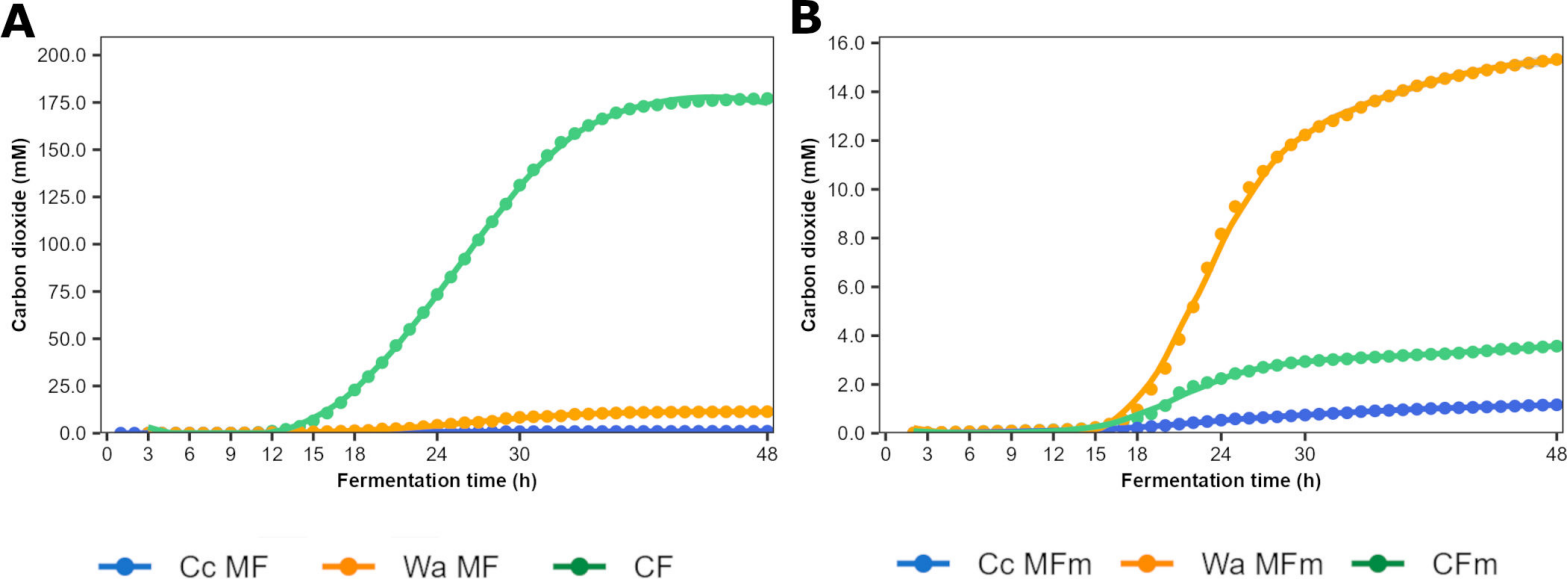
Carbon dioxide concentrations during *Coml. crustorum* LMG 23699 monoculture (Cc MF and Cc MFm), *W. anomalus* IMDO 010110 monoculture (Wa MF and Wa FMm), and coculture (CF and CFm) fermentation processes with both strains carried out in WSSM (**A**) and mWSSM (**B**).

The carbon dioxide concentrations at the end of the Wa MF processes carried out in WSSM and mWSSM were 11.4 mM and 15.3 mM, respectively (Fig. 4). Although *Coml. crustorum* LMG 23699 produced negligible carbon dioxide concentrations during the Cc MF processes, these concentrations were higher during the MF ones carried out in mWSSM than in those carried out in WSSM. Moreover, the carbon dioxide concentrations were higher during the CF processes carried in WSSM than during the Wa MF ones carried out in this medium, reaching concentrations of 177 mM at the end of the CF processes. In contrast, during the CF processes carried out in mWSSM, this increase in the carbon dioxide concentration did not occur.

### Carbon recovery

The carbon recovery was 1.20 ± 0.04 and 1.01 ± 0.14 during the Cc MF processes carried out in WSSM and mWSSM, respectively, corresponding to the total conversion of the carbohydrates into lactic acid. In the case of the Wa MF processes, the carbon recovery was 0.66 ± 0.04 and 0.99 ± 0.06 during the fermentation processes in WSSM and mWSSM, respectively. A carbon recovery lower than 1.00 in the case of the Wa MF processes carried out in WSSM corresponded with lower carbon dioxide concentrations measured compared with the theoretical ones that could be produced during the conversion of the carbohydrates into ethanol and carbon dioxide. The carbon recovery during the CF processes carried out in WSSM and mWSSM was 1.82 ± 0.11 and 1.80 ± 0.30, respectively. Carbon recoveries higher than 1.00 reflected a lactic acid production capacity that was higher than that possible from the carbohydrates available at the beginning of the fermentation processes, suggesting the use of additional carbon sources during these fermentation processes.

## DISCUSSION

The potential of *Coml. crustorum* LMG 23699 and *W. anomalus* IMDO 010110 as a new, mixed-strain starter culture for sourdough production by studying their interactions and the involvement of the capacity to produce esters with fruity notes was dealt with through mono- and co-culture fermentation processes in both WSSM and mWSSM during the present study. The latter medium contained ester precursor molecules and allowed us to give insights into the ester production capacity of the LAB strain, in addition to that of the yeast strain. Also, it showed the vulnerability of the yeast strain toward acidic stress.

The use of specific molecules in a fermentation medium to unravel hidden metabolite formation by specific strains has been shown before (5, 30, 41). It contributes to the unraveling of the metabolic potential of specific strains under conditions that do not always occur during common food fermentation processes. The chemical and physical fermentation conditions chosen, more specifically a carbohydrate content similar to that of wheat flour, wheat extract and yeast peptone to provide the necessary AAs, vitamins, and other growth factors, a fermentation temperature optimal for sourdough production with these starter culture strains (29), a free pH course, and cell densities similar to the ones found during sourdough production carried out with these strains (29) mimicked the sourdough food matrix and production conditions but reduced the dynamic complexity of a real sourdough production environment. This allowed us to study the interactions of the two microbial strains chosen, in the absence of an influencing background microbiota and without compromising the appliance of the results to the food matrix. The competitiveness of this mixed-strain starter culture for sourdough production could be attributed to the consumption of the carbohydrates characteristic for wheat flour till depletion, the use of other carbon sources for growth and maintenance, and faster acidification during the CF processes in WSSM compared with the MF ones. In addition, the differences in VOC profiles between the CF and MF processes could be of interest to produce sourdoughs and sourdough-containing baked products with novel flavor characteristics. Moreover, the higher carbon dioxide production during the CF processes compared with the MF ones could result in the desirable specific bread volumes.

Comparable growth courses of *Coml. crustorum* LMG 23699 during the MF and CF processes in WSSM, but an earlier start of the stationary phase of *W. anomalus* IMDO 010110 during the MF processes compared with the CF ones suggested an ammensalistic interaction. Similar growth courses of a LAB strain during MF and CF processes have been reported for other LAB-yeast combinations during simulated fermentation processes (42, 43) and sourdough production (42–44). Moreover, amensalism has been described before for other LAB-yeast combinations (5). However, another strain of *W. anomalus* negatively affected the cell density of a strain of *Lacp. plantarum* during the stationary phase when comparing CF and MF processes (45). The earlier start of the stationary phase of *W. anomalus* IMDO 010110 could be ascribed to its vulnerability toward acidic stress, given the presence of organic acids in mWSSM and the higher lactic acid production during the CF processes in mWSSM compared with those in WSSM. Nevertheless, based on these growth properties, the LAB and yeast strains of the consortium tested could optimally grow during the main phases of sourdough production and bread dough fermentation. Furthermore, the buffering capacity of the flour might reduce the acidic stress. Although the interaction between *Coml. crustorum* LMG 23699 and *W. anomalus* IMDO 010110 could also involve competition for the same limited resources, such as the carbohydrates glucose, fructose, and maltose, specific metabolite exchanges of, for instance, AAs could provide a survival advantage and enable the coexistence of both strains (46). The use of AAs resulted in higher carbon dioxide production, which could be in favor of dough leavening. In addition, the similar lactic acid and VOC production during the Cc MF processes carried out in both media, and during the CF processes carried out in mWSSM, during which the yeast cell density did not increase, suggested that the interaction between both strains, and, consequently, the desirable metabolic changes, required their active growth. Therefore, the sourdough maintenance conditions should be carefully optimized to keep stable and active microbial communities (47).

Yeast growth inhibition in both media occurred when the pH values were close to or lower than the pK_a_ values of the different organic acids present, namely, 3.8 for lactic acid (produced by *Coml. crustorum* LMG 23699 and added as ester precursor molecule), and 4.7 for acetic acid, 4.9 for propionic acid, 4.8 for butyric acid and pentanoic acid, 4.9 for hexanoic acid, heptanoic acid, octanoic acid, and decanoic acid, and 5.3 for dodecanoic acid (all added as ester precursor molecules). Indeed, non-dissociated organic acids can diffuse across the microbial cell membrane and exert an inhibitory effect (48, 49). In addition, the deleterious effect of acetic acid on yeast cells is more pronounced during the exponential growth phase compared with stationary-phase cells (48). Moreover, this acidic stress could lead to a change in microbial metabolism, such as the use of other carbon sources, as reflected by the carbon recovery data. Changes in the carbohydrate and lipid metabolism as well as the AA uptake, as a consequence of acidic stress, have been suggested before (48, 49).

Despite the similar pH values reached at the end of the Cc MF and CF processes carried out in WSSM, a faster drop of the pH during the CF processes indicated a competitive advantage of this LAB-yeast consortium over the background microbiota when they were applied as a mixed-strain starter culture for sourdough production. In contrast, different final pH values during MF and CF processes have been reported before for other LAB-yeast combinations (43). The pH drop during all fermentation processes of the present study was linked to the production of lactic acid, the latter being produced in higher quantities during the CF processes compared with the Cc MF ones, which was also the cause of the faster pH drop during the former fermentation processes. The slight production of lactic acid during the Wa MF processes yielded concentrations that were similar to those reported before for other strains of this yeast species (50, 51). Moreover, the further consumption of lactic acid during the Wa MF processes has been described for strains of other yeast species too (8). The higher lactic acid concentrations during the CF processes could be the result of the use of other energy sources than carbohydrates, such as AAs, as reflected by the increase of the AA content during the Cc MF processes but not during the CF ones carried out in WSSM. For example, alanine and asparagine could be converted into pyruvate and further into lactic acid (52). The faster and higher lactic acid production during the CF processes compared with the MF ones could affect the specific bread volume when a sourdough started with this mixed-strain starter culture would be used for sourdough bread production, as the addition of lactic acid to reach a bread dough pH value of 4.5 increases the specific bread volume compared with a control bread without organic acid addition (53). The concentrations of ethanol produced during the Wa MF processes carried out in WSSM, those of glycerol produced during the exponential phase, and those of arabitol produced during the stationary phase were similar to those described for other *W. anomalus* strains under anaerobiosis (40, 54, 55). The lower ethanol production during the CF processes carried out in WSSM, as well as during the Wa MF processes carried out in mWSSM, could indicate a change from a fermentative to a respiratory metabolism, most likely as a result of different needs of ATP under acidic conditions (44), or the use of non-fermentable carbon sources, such as AAs, which do not result in ethanol production, as a result of the competition for limited carbohydrates.

The different VOC profiles that resulted from the CF processes carried out in WSSM compared with the MF ones could be of interest for the flavor and quality of sourdoughs produced with this mixed-strain starter culture. Higher concentrations of VOCs during the CF processes compared with the MF ones have been reported before for phenolic compounds (5). The biosynthesis of VOCs linked to the Ehrlich pathway, such as phenylacetaldehyde (sweet notes), and the esters allyl acetate (honey notes), ethyl acetate (sweet notes), and ethyl pyruvate (caramel and citrus notes), might be the response of the yeast strain to the acidic stress encountered (7, 28). Hence, the use of the mixed-strain starter culture of the present study for sourdough production could lead to fruitier bread crumbs. The production of different and more flavor-active compounds by yeast strains in the presence of LAB strains has been reported before (26), but the ester production by the LAB strain could have a role too, especially concerning the biosynthesis of ethyl lactate (creamy notes) (32). The addition of ester precursors influenced the bacterial and yeast metabolism, leading to different VOC profiles in mWSSM compared with WSSM. Particularly, the addition of ester precursors allowed the formation of ethyl lactate during the Cc MF processes, showing that the limitation for the biosynthesis of this ester was not the inability of *Coml. crustorum* LMG 23699 to produce esters but the absence of precursor molecules, *in casu* ethanol (32). The chemical formation of ethyl lactate has been reported in uninoculated mWSSM fermentation processes (32), but the concentrations were lower than those reported in the present study. However, during the CF processes, this ester was produced in both media, indicating that starter cultures composed of yeast and LAB strains could result in the formation of esters that are not synthesized if only the LAB strain is used during sourdough production.

The differences in carbon dioxide concentrations produced during the MF and CF processes, as measured in the outlet of the fermentors, were remarkable. The low concentrations of carbon dioxide produced during the Wa MF processes corresponded to an intrinsic characteristic of *W. anomalus* (25). However, the lower carbon dioxide concentrations measured in the Wa MF processes compared with those theoretically estimated, based on the ethanol produced, could be ascribed to the retention of carbon dioxide in the fermentation medium as a result of pH values that were close to 6.0 during these processes. The higher carbon dioxide concentrations produced during the CF processes compared with the MF ones, despite the fact that *Coml. crustorum* LMG 23699 is a homofermentative LAB species, could be ascribed to a higher carbon dioxide production by *W. anomalus* IMDO 010110, but not through ethanol production. This could be linked to an increased AA uptake and further conversion, including decarboxylation, by the yeast strain as a response to the acidic stress, also resulting in the higher production of higher aldehydes and esters as mentioned above. This increased AA uptake has been described for ethanol stress in another strain of the same species (56). Similarly, AA transporters are upregulated in *S. cerevisiae* in the presence of *Oenococcus oeni* (57). In addition, the higher acetoin production during the CF processes contributed to the higher carbon dioxide production. The increased carbon dioxide production during the CF processes of the present study could lead to a better leavening capacity of the mixed-strain starter culture investigated, as has been reported before for certain combinations of homofermentative LAB and yeast strains (16, 26, 58).

Finally, it has to be noticed that the LAB-yeast interactions described in the present study cannot be extrapolated to other strains of *Coml. crustorum* and *W. anomalus*, as these interactions are strain-specific (6, 22). Therefore, the potential for a mixed-strain starter culture for sourdough production is only for this specific combination of LAB and yeast strains. Further research based on gene expression could give deeper insights into the interactions established between *Coml. crustorum* LMG 23699 and *W. anomalus* IMDO 010110, as well as the genes implicated in flavor formation and its potential impact on these interactions during the CF processes.

In conclusion, the fermentation processes carried out in WSSM unraveled the competitiveness of *Coml. crustorum* LMG 23699 and *W. anomalus* IMDO 010110 as mixed-strain starter culture, as well as their interactions and ester formation potential by each of the strains. Although competitiveness for the same carbon sources occurred, the interactions of these two strains resulted in a faster pH decrease, which could give a competitive advantage to this consortium and the production of desirable metabolites, such as acetoin and esters with buttery and fruity notes, respectively, for the production of sourdough-containing baked products. In addition, the Cc MF processes carried out in mWSSM confirmed the ethyl lactate (creamy notes) production capacity by *Coml. crustorum* LMG 23699.

## MATERIALS AND METHODS

### Strains and inoculum build-up

The LAB strain *Coml. crustorum* LMG 23699 (59) and the yeast strain *W. anomalus* IMDO 010110 (29) were used throughout this study. They originated from Belgian artisan wheat sourdough, and a Type 3 sourdough production started with the LAB strain, respectively. These strains were kept at −80°C in WSSM (60), supplemented with 25% glycerol (Sigma-Aldrich, Saint-Louis, Missouri, USA) as a cryoprotectant, at the laboratory collection of the research group of Industrial Microbiology and Food Biotechnology (IMDO-VUB). For inoculum build-up, they were grown on de Man-Rogosa-Sharpe (mMRS-5) agar medium (LAB strain; 61) or yeast extract-peptone-glucose (YPG) agar medium (yeast strain; 30) by incubation at 30°C for 48 h. One colony was then transferred to 5 mL of WSSM and incubated at 30°C for 24 h. Before the start of each fermentation process, 0.2 mL of this preculture was transferred to 20 mL of the medium used to carry out the actual fermentation process and incubated at 30°C for 12 h (LAB strain) or 18 h (yeast strain). This culture was used to start the fermentation processes.

### Fermentation processes

#### Media

Both WSSM (60) and modified WSSM (mWSSM; 32) were used to carry out the fermentation processes. WSSM contained 0.5 g/L of fructose (Merck, Darmstadt, Germany), 0.5 g/L of glucose (Merck), 10.0 g/L of maltose (Merck), 2.0 g/L of sucrose (Merck), 12.0 g/L of wheat peptone (Merck), 12.0 g/L of yeast extract (Oxoid, Basingstoke, Hampshire, UK), 4.0 g/L of dipotassium phosphate (Merck), 0.2 g/L of magnesium sulfate (Merck), 0.05 g/L of manganese (II) sulfate (Merck), 4.0 g/L of potassium phosphate (Merck), 1.0 mL/L of Tween 80 (Sigma-Aldrich), and 1 mL/L of a vitamin solution encompassing cobalamin, folic acid, nicotinamide, pantothenic acid, pyridoxal phosphate, and thiamine (0.2 g/L each). In addition, mWSSM contained an ester precursor solution consisting of ethanol (final concentration of 1.0%, vol/vol), acetic acid (final concentration of 0.5%, vol/vol), other organic acids (propionic acid, butyric acid, pentanoic acid, hexanoic acid, decanoic acid, and lactic acid; final concentrations of 5.0 mg/L each), and higher alcohols (1-propanol, 1-butanol, 1-pentanol, 1-hexanol, and isoamyl alcohol; final concentrations of 5.0 mg/L each).

#### Mono- and co-culture fermentation processes and sampling

Mono- and co-culture fermentation processes (referred to as MF and CF, respectively) were carried out in both WSSM and mWSSM for 48 h, making use of Biostat B-DCU fermentors (Sartorius, Göttingen, Germany) equipped with temperature (adjusted at 30°C) and agitation (100 rpm) control. Each fermentation process was carried out in triplicate. The fermentors were first filled with 1.8 L of WSSM (pH of 6.7) without carbohydrates, followed by sterilization at 121 ˚C and 2.1 bar for 20 min. A 200 mL solution of the appropriate carbohydrates (glucose, fructose, sucrose, and maltose) was sterilized under the same conditions separately and added aseptically to the fermentors just before the start of the fermentation processes. In addition, for the fermentation processes carried out in mWSSM, the ester precursor solution was added aseptically to the fermentor, and the pH was adjusted to 6.7 before the start of the fermentation processes. Finally, to start each fermentation process, the appropriate strain(s) was(were) inoculated at 1% (vol/vol), using the preculture(s) mentioned above.

Samples were taken after inoculation (referred to as time point 0) and after 3, 6, 9, 12, 15, 18, 24, 30, and 48 h of fermentation, to follow up the microbial growth, pH, and metabolite production and conversion dynamics.

### Microbial growth

The microbial growth was monitored through measurements of the colony-forming units (CFUs, log [CFU/mL]), optical density at 600 nm (OD_600_), and cell dry mass (CDM) in the samples taken. The CFUs were determined by plating 10-fold serial dilutions of each sample in saline (0.85% [m/vol] NaCl; Merck) on mMRS-5 agar medium (LAB strain) and YPG agar medium (yeast strain), supplemented with 0.4 g/L of cycloheximide (Sigma-Aldrich) and 0.005 g/L of amphotericin B (Sigma-Aldrich) or 0.2 g/L of chloramphenicol (Sigma-Aldrich), respectively. The agar media were incubated at 30°C for 48 h. The data obtained were fitted to the logistic equation (62). The OD_600_ was measured with a spectrophotometer (Genesys 20; Sigma-Aldrich). Ultrapure water (MilliQ; EMD Millipore, Burlington, Massachusetts, USA) was used as a blank. For quantification of the CDM, 15 mL of fermentation sample was filtered through a cellulose nitrate membrane filter (VWR International, Darmstadt, Germany), and the washed retentate was oven-dried at 105°C for 24 h.

### pH

The pH of each fermentation sample was measured immediately, using an InoLab 720 pH meter (WTW, Weilheim, Germany).

### Metabolite target analysis

Fermentation sample processing and preparation were performed as described previously (47), except as stated otherwise. All measurements were performed in triplicate.

#### Carbohydrates

The concentrations of carbohydrates (fructose, glucose, maltose, and sucrose) were determined *via* HPAEC-PAD, using an ICS 5000 chromatograph equipped with a CarboPac PA20 column in combination with an ED-40 PAD detector (Dionex, Sunnyvale, California, USA), as described previously (47).

#### Lactic acid and other organic acids

Citric acid, lactic acid, malic acid, oxalic acid, and succinic acid were quantified through ultra-performance liquid chromatography with tandem mass spectrometry detection (UPLC-MS/MS), using a Waters Acquity UPLC system (Waters, Milford, Massachusetts, USA) equipped with a HSS T3 column (Sigma-Aldrich) in combination with a triple-quadrupole (TQ) tandem mass spectrometer (Waters), as described previously (63). Salicylic acid (0.02 g/L; Sigma-Aldrich) was used as an internal standard (IS). Sample processing and preparation were performed as described previously (63).

Also, the ratio of D-lactic acid to L-lactic acid was determined by UPLC-MS/MS, by means of the same equipment as mentioned above, using an Astec Chirobiotic T column v04 (Sigma-Aldrich) and salicylic acid as IS, as described previously (64).

#### Sugar alcohols

The concentrations of sugar alcohols (arabitol and glycerol) were determined using high-performance anion exchange chromatography with pulsed amperometric detection (HPAEC-PAD), using an ICS 5000 chromatograph equipped with a CarboPac MA1 column in combination with an ED-40 PAD detector (Dionex), as described previously (65).

#### Ethanol and acetic acid

The concentrations of ethanol and acetic acid were determined using gas chromatography with flame ionization detection (GC-FID), using a Trace 1310 gas chromatograph (Thermo Fisher Scientific, Waltham, Massachusetts, USA) equipped with a DBwax-UI column (Agilent Technologies, Santa Clara, California, USA) in combination with an Instant connect FID detector (Thermo Fisher Scientific), as described previously (47). For the fermentation samples of the processes carried out in mWSSM, 1-heptanol instead of 1-butanol was used as IS.

#### Amino acids and biogenic amines

Free proteinogenic AAs, citrulline, gamma-aminobutyric acid (GABA), ornithine, and biogenic amines (agmatine, cadaverine, histamine, 2-phenylethylamine, putrescine, spermidine, spermine, tryptamine, and tyramine) were quantified by UPLC-MS/MS, using the same equipment as that used for the quantification of organic acids, as described previously (66). Fermentation sample processing and preparation were performed as described previously (66).

#### Volatile organic compounds

Selected VOCs were quantified by liquid injection GC with TQ tandem mass spectrometry (LI-GC-TQ-MS), using a Trace 1300 gas chromatograph equipped with a DBwax-UI column (Agilent) in combination with a TSQ 8000 EVO TQ mass spectrometer (Thermo Fisher Scientific), as described previously (67). Fermentation sample processing and preparation were performed as described previously (47). Only the samples after inoculation (0), and after 6, 9, 12, 24, 30, and 48 h of fermentation were analyzed.

#### Carbon dioxide

Carbon dioxide concentrations were measured online in the outlet of the fermentors (every 30 min) by GC with thermal conductivity detection (CompactGC; Interscience, Breda, the Netherlands), as described previously (68). Briefly, the analytical channel used for monitoring the carbon dioxide concentrations was equipped with a carrier gas module (helium at 70 kPa), an injection valve, a column oven (consisting of a 2 m PoraBOND Q guard column followed by a 10 m main column of the same type [Varian, Palo Alto, California, USA]), and a thermal conductivity detector. The transfer of the gas produced in the fermentors to the CompactGC was done using a sample pump coupled to a selection valve. The temperature valve was set at 60°C, and 20 µL was injected at atmospheric pressure using a split flow of 5 mL/min and a reference flow of 1 mL/min. The column temperature was set at 60°C and the detector temperature at 110°C. The calibration was done with calibration gas mixtures (Saphir calibration gases; Air Liquide, Paris, France) containing 2,000 and 20,000 ppm of carbon dioxide. The CompactGC was controlled with the CGC Editor 1.53 software package (Interscience), and peak integration was done with the EZChrom Elite 3.2 software package (Agilent).

### Carbon recovery

The carbon recovery during each fermentation process was calculated as the ratio of the carbon produced (carbon dioxide, ethanol, lactic acid, and sugar alcohols) plus the carbon present in the form of non-consumed carbohydrates to the carbon present in the form of carbohydrates at the beginning of the fermentation processes.

### Statistical analysis

All statistical tests were performed with RStudio software (version 2022.07.1; 69). To assess statistical differences among the different fermentation processes carried out, the normality of the data was checked by a Shapiro test. Statistical differences between the two types of fermentation processes were assessed by a Wilcoxon Rank Sum test. A *P*-value < 0.05 was considered significant.

A principal component analysis (PCA) was carried out to reveal clusters among the different fermentation processes, using the VOCs measured by means of LI-GC-TQ-MS. The PCA analysis was performed using the ggfortify package (v0.4.14; 70).

## Data Availability

Data will be made available on request.

## SUPPLEMENTAL MATERIAL

Supplemental figures aem.01325-25-s0001.pdf Figures S1 to S5.

ASM does not own the copyrights to Supplemental Material that may be linked to, or accessed through, an article. The authors have granted ASM a non-exclusive, world-wide license to publish the Supplemental Material files. Please contact the corresponding author directly for reuse.
