# Supplementary material for: Coculture fermentation processes in wheat sourdough simulation media with Companilactobacillus crustorum LMG 23699 and Wickerhamomyces anomalus IMDO 010110 reflect their competitiveness and desirable traits for sourdough and sourdough bread production
Source: Appl Environ Microbiol. 2025 Aug 12;91(9):e01325-25. doi: 10.1128/aem.01325-25 (PMC12442410; doi:10.1128/aem.01325-25)
Supplement: Supplemental figures — Figures S1 to S5. [file aem.01325-25-s0001.pdf]

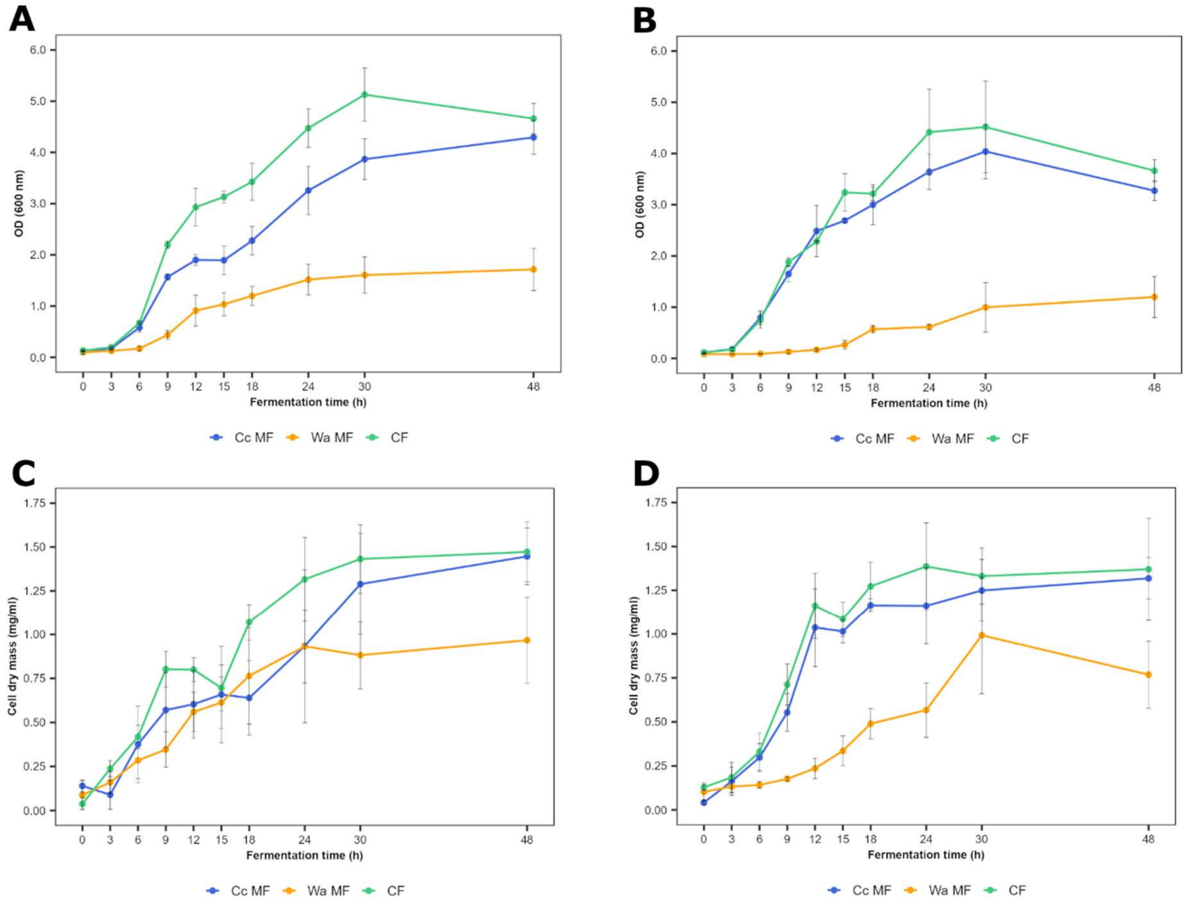

Fig. S1. Microbial growth course of *Companilactobacillus crustorum* LMG 23699 (Cc) and *Wickerhamomyces anomalus* IMDO 010110 (Wa) in WSSM (left) and mWSSM (right) during monoculture (MF) and coculture (CF) fermentation processes, optical density at 600 nm (**A** and **B**), and cell dry mass (**C** and **D**). The averages and standard deviations of three fermentation processes are shown.

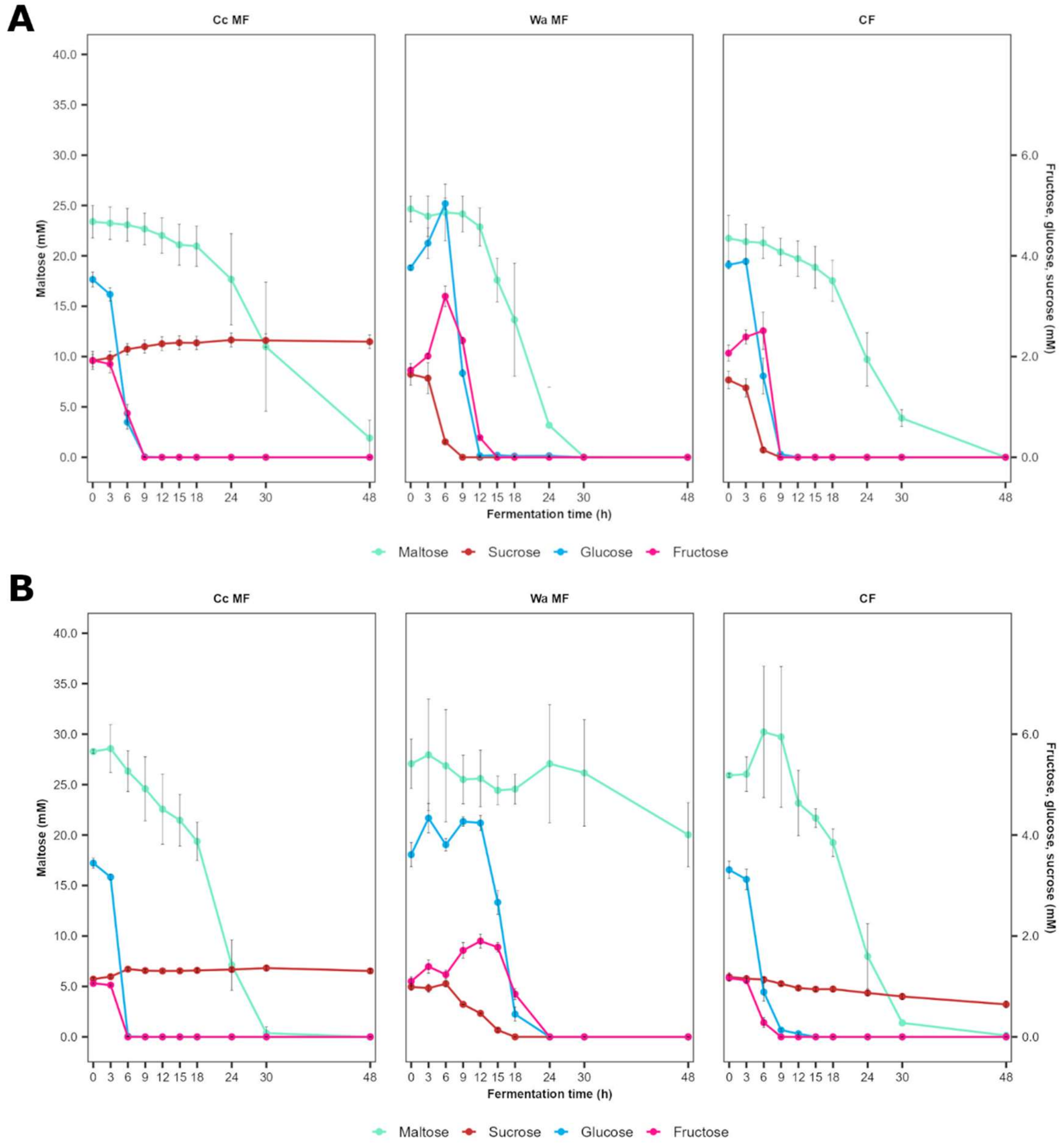

Fig. S2. Carbohydrate concentration dynamics during *Companilactobacillus crustorum* LMG 23699 monoculture (Cc MF), *Wickerhamomyces anomalus* IMDO 010110 monoculture (Wa MF), and coculture (CF) fermentation processes with both strains carried out in WSSM (**A**) and mWSSM (**B**). The averages and standard deviations of three fermentation processes are shown.

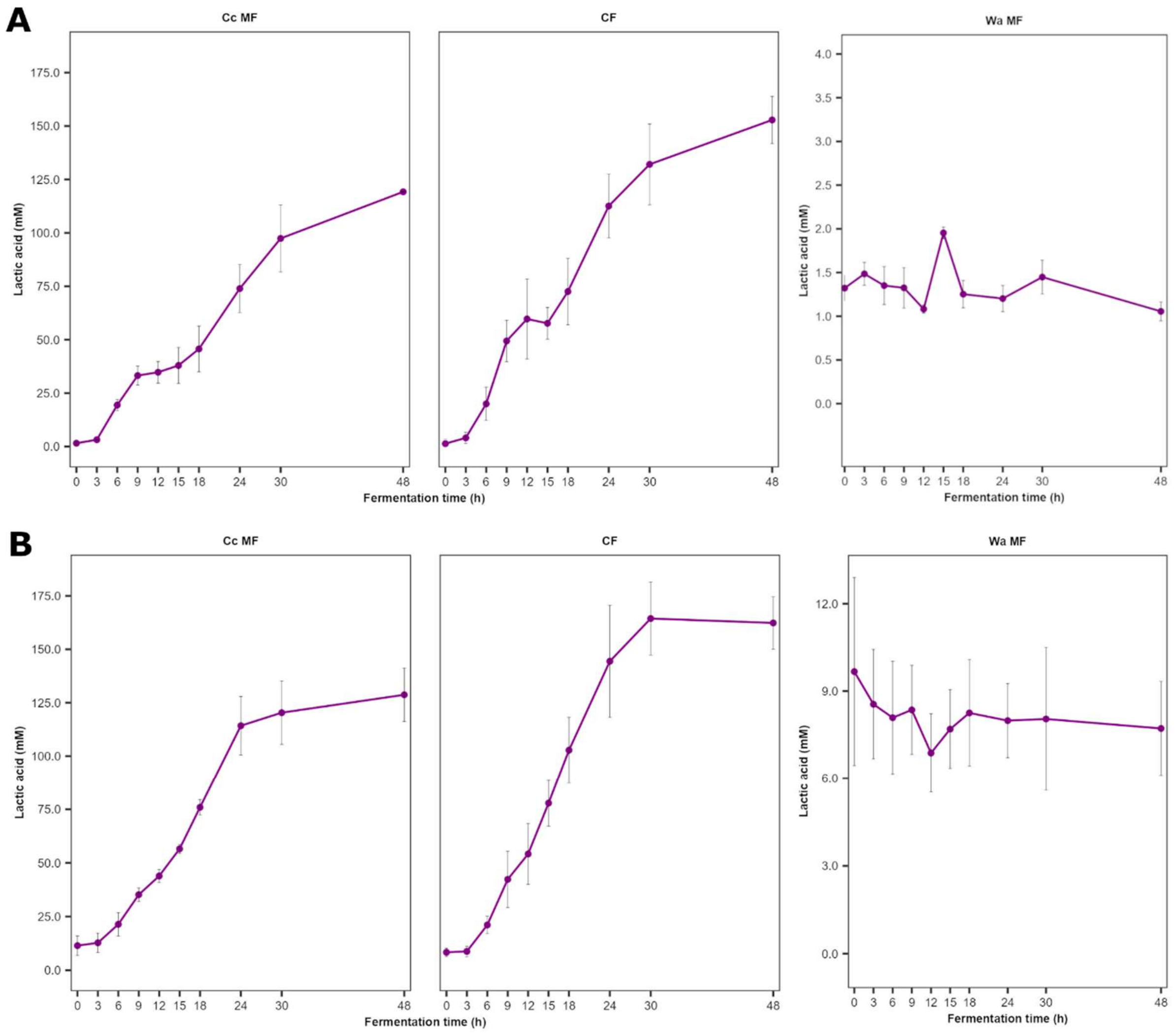

Fig. S3. Lactic acid concentration dynamics during *Companilactobacillus crustorum* LMG 23699 monoculture (Cc MF), *Wickerhamomyces anomalus* IMDO 010110 monoculture (Wa MF) and coculture (CF) fermentation processes with both strains carried out in WSSM (**A**) and mWSSM (**B**). The averages and standard deviations of three fermentation processes are shown.

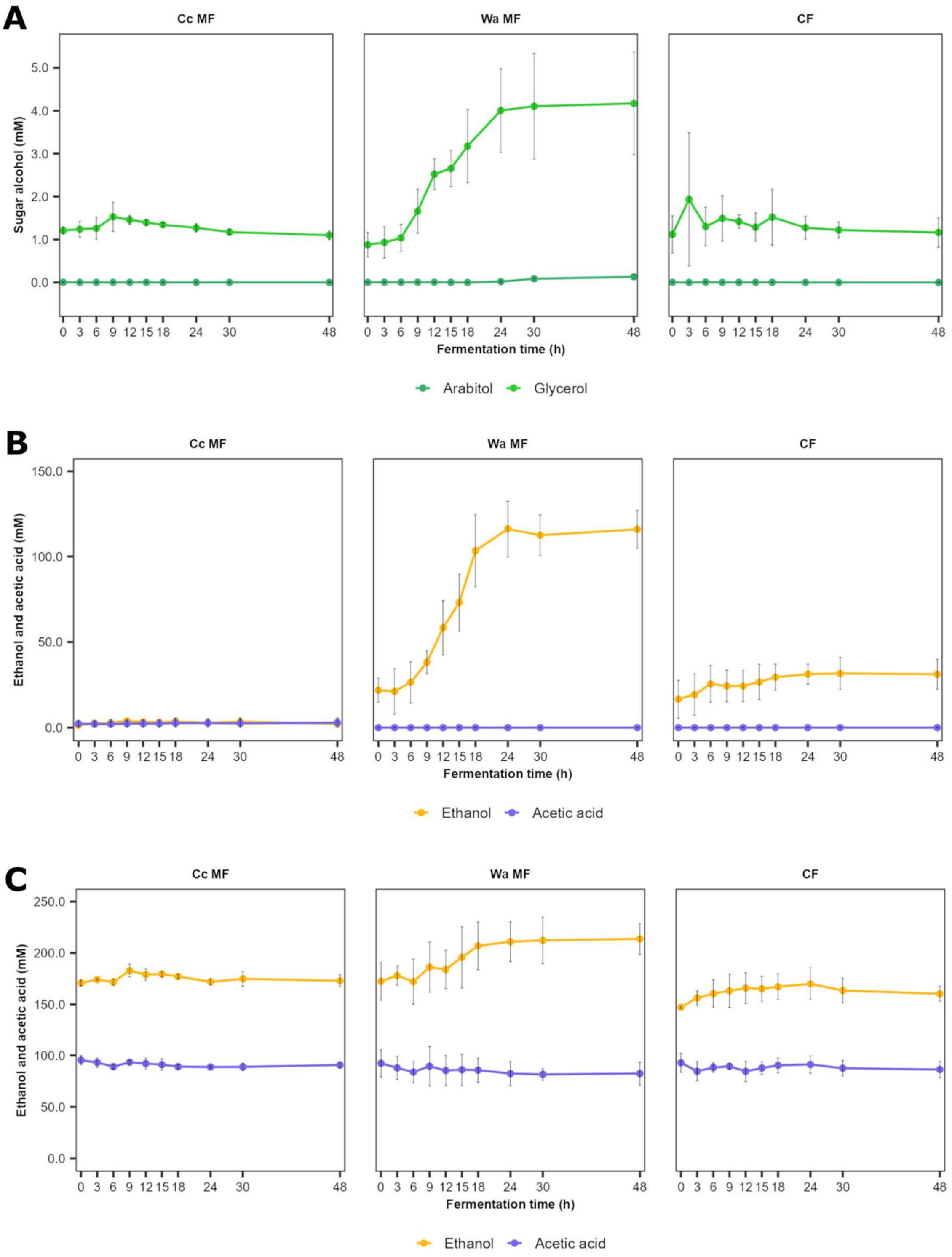

Fig. S4. Sugar alcohol (**A**) and ethanol and acetic acid (**B** and **C**) concentration dynamics during *Companilactobacillus crustorum* LMG 23699 monoculture (Cc MF), *Wickerhamomyces anomalus* IMDO 010110 monoculture (Wa MF), and coculture (CF) fermentation processes with both strains carried out in WSSM (A and B) and mWSSM (C). The averages and standard deviations of three fermentation processes are shown.

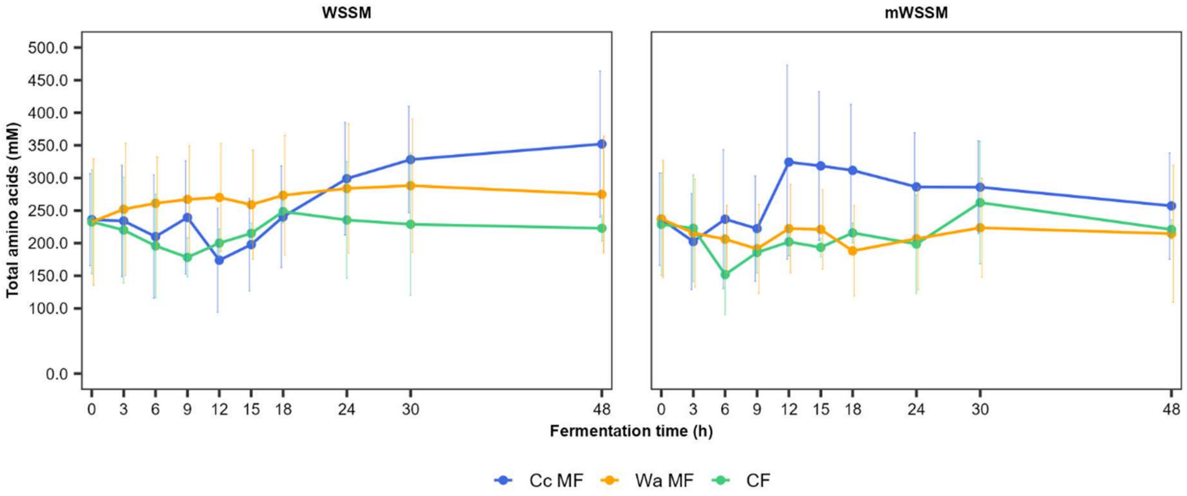

Fig. S5. Free amino acid concentration dynamics during *Companilactobacillus crustorum* LMG 23699 monoculture (Cc MF), *Wickerhamomyces anomalus* IMDO 010110 monoculture (Wa MF) and coculture (CF) fermentation processes with both strains carried out in WSSM (left) and mWSSM (right). The averages and standard deviations of three fermentation processes are shown.
